# Current status of traumatic spinal cord injury caused by traffic accident in Northern China

**DOI:** 10.1038/s41598-022-16930-9

**Published:** 2022-08-16

**Authors:** Yao Wu, Zhenrong Zhang, Fangyong Wang, Wenjing Wang

**Affiliations:** 1 School of Rehabilitation Medicine, Capital Medical University, Beijing, China; 2 Department of Spine Surgery, Beijing Bo’ai Hospital, China Rehabilitation Research Center, No. 10, Jiaomen North Road, Fengtai District, Beijing, 100068 China; 3 Department of Occupational Therapy, Beijing Bo’ai Hospital, China Rehabilitation Research Center, Beijing, China

**Keywords:** Disease prevention, Neurological disorders

## Abstract

The study aims to investigate the characteristics of traumatic spinal cord injury (TSCI) caused by motor vehicle collisions (MVCs). The study included 649 cases who experienced MVC-induced TSCI. The mean age was 37.3 years old, ranging from 1 to 82 years old. The male-to-female ratio was 2.7:1. We extracted data of gender, age, occupation, neurological level of injury, fracture level, complications, vehicle type, accident type and other features. The results showed that the most common vehicles involved in accidents were passenger cars (65.1%). Collision was the leading cause of MVCs (35.8%). The lesion level was cervical in 51.6% and thoracic in 42.2%. The most common fracture levels in drivers and passengers were C3–C7, while those in pedestrians were T11–L2. The frequency of tetraplegia (51.6%) was slightly higher than paraplegia (48.4%), and cases with tetraplegia with incomplete injury accounted for 61.2%. MVC-induced TSCI has unique clinical features. Collision was the most common cause. People sitting in cars were more likely to suffer from cervical fractures, while thoracolumbar fractures were more common in pedestrians. Tetraplegic cases were mainly incomplete injuries, while paraplegic cases were mainly complete injuries.

## Introduction

Traumatic spinal cord injury (TSCI) remains a serious public health problem, leading to different degrees of neurological deficits under the level of injury^1^. There is no definitive cure for TSCI, and prevention is particularly important. The analysis of epidemiological characteristics is helpful for improving the awareness of risk factors and reducing the incidence of TSCI caused by motor vehicle collisions (MVCs).

The overall global incidence of TSCI is 100.5 cases per million, resulting in an estimated 768,473 new cases of TSCI annually worldwide^2^. In China, the incidence varies in different regions. Studies have demonstrated that the annual incidence of TSCI was 73.0 cases per million in Taiwan (2000–2003)^3^. In addition, *Ning* et al. reported an incidence rate of 23.7 per million between 2004 and 2008 in Tianjin^4^. Li et al. showed that the incidence of acute TSCI per million population each year was 60.6 during 2002 in Beijing, which is much higher than that of other regions^5^.

MVCs are the leading causes of TSCI in many countries^6,7^. Vehicle type, age, behavior of drivers, accident type, weather, and traffic regulations affect the rate of spinal injuries to different extents^8^. However, traffic accidents resulting from the violation of road traffic safety regulations due to drivers’ negligence are largely preventable. Many studies have been limited by small samples, insufficient data, and selection bias^9,10^. Different to what has been published in our rehabilitation center, we included all cases with TSCI caused by traffic accidents whether or not they were residents of Beijing at the time of injury and excluded other causes of TSCI^11^. Therefore, it is necessary to provide comprehensive data on TSCIs resulting from MVCs. The goals of this study were to investigate the features of the hospitalized population with MVC-induced TSCI from January 1, 2010 to December 31, 2019, and provide effective advice for preventive measures.

## Materials and methods

### Study design

The China Rehabilitation Research Center (CRRC) is the largest national comprehensive rehabilitation centers in China, and is responsible for high quality treatment of TSCI. The patients in CRRC came mainly from the northern provinces of China including Beijing, Tianjin, Hebei, Shanxi, Inner Mongolia, Liaoning, Jilin, Heilongjiang, Shandong, Henan, Tibet, Shaanxi, Gansu, Qinghai, Ningxia and Xinjiang, which can represent the characteristics of TSCI in northern China. This is a retrospective study of 649 cases with TSCI who were involved in traffic accidents and then admitted to CRRC. Their medical records were reviewed and assessed by two independent researchers.

### Characteristics of participants

According to the International Spinal Cord Injury Core Data Set (version 2.0) published in 2017, these cases were divided into five age groups: 1–14, 15–29, 30–44, 45–59, and ≥ 60 years old, so as to find out the characteristic of pediatric and adult populations with SCI caused by traffic accident^12^. Four hundred and twenty-one cases were admitted to CRRC within 6 months after injury, which accounted for 64.9%. The severity of injury was recorded using the American Spinal Injury Association classification and divided into complete injury (Grade A) and incomplete injury (Grade B, C, D)^13^. The neurological level of injury was classified as cervical, thoracic, lumbar, sacral or cauda equina, which was based on the International Standards for Neurological Classification of Spinal Cord Injury. We try our best to find the neurological function of the children both by their description and respond to stimulation. And we do seek to other testing methods to ensure the reliability of the neurological examination in subjects under 14 years of age, as *Manworren* et al. reported before^14^. Three approaches including physiological (how the child’s body reacts), behavioral (how the child behaves), and self-report (what the child says) indicators were used to determine children’s neuralgia. With pre-verbal and non-verbal children, behavioral pain assessment tools were used. Behavioral pain assessment tools are commonly used, valid and reliable proxy scales, including Children’s Hospital of Eastern Ontario Pain Scale (CHEOPS) and Paediatric Pain Profile^15,16^. Payment method was divided into own expense and others (Basic Medical Insurance for Urban Residents and Urban Employees, New Rural Cooperative Medical Insurance, other commercial medical insurance, and free medical service). Vehicle type included non-motor vehicle (bicycle, electric assist bicycle, and tricycle), motorcycle, passenger car (car, off-road vehicle, and multi-purpose vehicle), commercial vehicle (bus, truck, and ambulance), and others (tramcar, trailer, forklift, tanker, and excavator). The causes of the traffic accidents were divided into vehicle collision, rear-end, high vehicle fall (fall from a height of ≥ 1 m), rollover, struck objects (tree, pole, and rail), flat tire, and fall from the vehicle.

### Statistical analysis

We collected data on gender, age, marital status, payment method, length of stay, total cost, vehicle type, and accident type as demographic variables and occupation, neurological level of injury, the American Spinal Injury Association impairment scale on admission, fracture level, surgical approach, degeneration of vertebrae, complications, and associated injuries as clinical variables.

All statistical analyses were performed using Statistical Product and Service Solutions (version 25.0 Inc., Chicago, IL, USA) and Microsoft Excel (Microsoft Corporation, Redmond, WA, USA). Continuous variables between regions were determined by the independent Student’s t-test, whereas categorical variables were analyzed by performing a between-group chi-squared test and Fisher’s exact test. The comparison of the constituent ratios of two or more groups was mainly selected chi square test. When the sample size in the R × C contingency table was less than 40 or the theoretical frequency of one grid was less than 1, it was easy to make the first type of statistical error, so Fisher's exact test should be used. Descriptive statistics (mean, standard deviations, frequency, and percentage) were used to describe demographic and medical characteristics of study participants caused by traffic accident. Statistical significance was set at *P* < 0.05.

### Ethical statements

The ethics committee of China Rehabilitation Research Center approved the study protocol. All methods were carried out in accordance with relevant guidelines and regulations (Declaration of Helsinki). Informed consent was obtained from all participants or a parent and/or legal guardian those who are under 16.

## Results

### Age and gender

**Figure 1:**
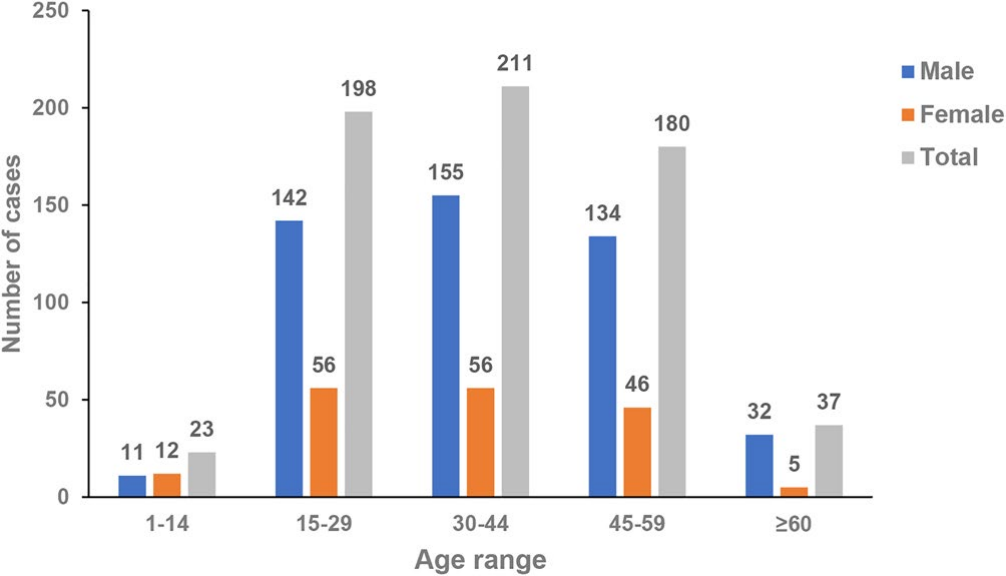
Age distribution of all 649 cases.

Age and gender distributions are shown in Table 1. The study included 474 males and 175 females with a mean age of 37.3 (14.1) years old (men 38.4 (14.1), women 34.4 (13.8); t = 3.274, *P* = 0.001), with a range of 1–82 years old. The male/female ratio was 2.7:1. The age distribution showed a peak at the 30–44 age group, accounting for 32.5%, followed by the 15–29 age group (30.5%). Cases in the ≤ 14 age group had the lowest sex ratio of 0.9. Cases in the ≥ 60 age group had the largest sex ratio of 6.4 (Fig. 1).

**Table 1:** Characteristics of 649 cases according to different age range groups.

Variables	Age group					Total	Statistics	*P*
	1–14	15–29	30–44	45–59	≥ 60			
Frequency (%)	23 (3.5%)	198 (30.5%)	211 (32.5%)	180 (27.7%)	37 (5.7%)	649 (100%)		
**Gender**							χ2 = 11.197	0.024
Male	11 (47.8%)	142 (71.7%)	155 (73.5%)	134 (74.4%)	32 (86.5%)	474 (73%)		
Female	12 (52.2%)	56 (28.3%)	56 (26.5%)	46 (25.6%)	5 (13.5%)	175 (27%)		
**Level of the injury**								
Cervical	2 (8.7%)	84(42.4%)	116(55%)	104 (57.8%)	29 (78.4%)	335 (51.6%)	χ2 = 37.966	< 0.001
Thoracic	20 (87%)	98(49.5%)	85(40.3%)	64 (35.6%)	7 (18.9%)	274 (42.2%)	χ2 = 35.001	< 0.001
Lumbar	0 (0%)	10(5.1%)	4(1.9%)	6 (3.3%)	1 (2.7%)	21 (3.2%)	F = 3.247	0.452
Conus	1 (4.3%)	6(3%)	6 (2.8%)	6 (3.3%)	0 (0%)	19 (2.9%)	F = 1.314	0.843
**ASIA grade**								
A	14 (60.9%)	125 (63.1%)	118 (55.9%)	90 (50%)	9 (24.3%)	356 (54.9%)	χ2 = 21.550	< 0.001
B	4 (17.4%)	27 (13.6%)	29 (13.7%)	24 (13.3%)	5 (13.5%)	89 (13.7%)	χ2 = 0.287	0.991
C	3 (13.0%)	28 (14.1%)	32 (15.2%)	37 (20.6%)	17 (45.9%)	117 (18%)	χ2 = 23.873	< 0.001
D	2 (8.7%)	18 (9.1%)	31 (14.7%)	29 (16.1%)	6 (16.2%)	86 (13.3%)	χ2 = 5.341	0.254
**Marital status**							F = 357.678	< 0.001
Married	0 (0%)	66 (33.3%)	198 (93.8%)	177 (98.3%)	37 (100%)	478 (73.7%)		
Unmarried	23 (100%)	132 (66.7%)	13 (6.2%)	3 (1.7%)	0 (0%)	171 (26.3%)		
**Payment method**							χ2 = 11.235	0.024
Own expense	15 (65.2%)	142 (71.7%)	128 (60.7%)	104 (57.8%)	19 (51.4%)	408 (62.9%)		
Others	8 (34.8%)	56 (28.3%)	83 (39.3%)	76 (42.2%)	18 (48.6%)	241 (37.1%)		
**Length of stay (day)**							χ2 = 2.880	0.578
0–364	19 (82.6%)	163 (82.3%)	166 (78.7%)	149 (82.8%)	33 (89.2%)	530 (81.7%)		
≥ 365	4 (17.4%)	35 (17.7%)	45 (21.3%)	31 (17.2%)	4 (10.8%)	119 (18.3%)		
**Complications**							χ2 = 8.849	0.065
Yes	13 (56.5%)	124 (62.6%)	134 (63.5%)	129 (71.7%)	30 (81.1%)	430 (66.3%)		
No	10 (43.5%)	74 (37.4%)	77 (36.5%)	51 (28.3%)	7 (18.9%)	219 (33.7%)		
**Total cost (million)**							χ2 = 4.328	0.363
0–0.1	14 (60.7%)	88 (44.4%)	96 (45.5%)	92 (51.12%)	15 (40.5%)	305 (47%)		
≥ 0.1	9 (39.1%)	110 (55.6%)	115 (54.5%)	88 (48.9%)	22 (59.5%)	344 (53%)

Most cases were married (*n* = 478, 73.7%) and the percentage of unmarried cases was 16.3%. Those who had been hospitalized for more than one year were mainly aged 30–44 years. The ≥ 60 age group had a higher total cost, which may have been attributed to more complications in older people (81.1%).

### Occupations

The occupations of those with SCI were divided into employed (26.1%), unemployed (16%), peasants (9.2%), self-employed (6.5%), students (5.4%), civil servants (4%), retired (3.2%), active service soldiers (1.8%), freelancers (1.8%), and enterprise administrators (0.8%). The employed included workers (12.5%), staff (10.8%) and professional skilled workers (2.8%).

### Neurological level of injury

**Figure 2:**
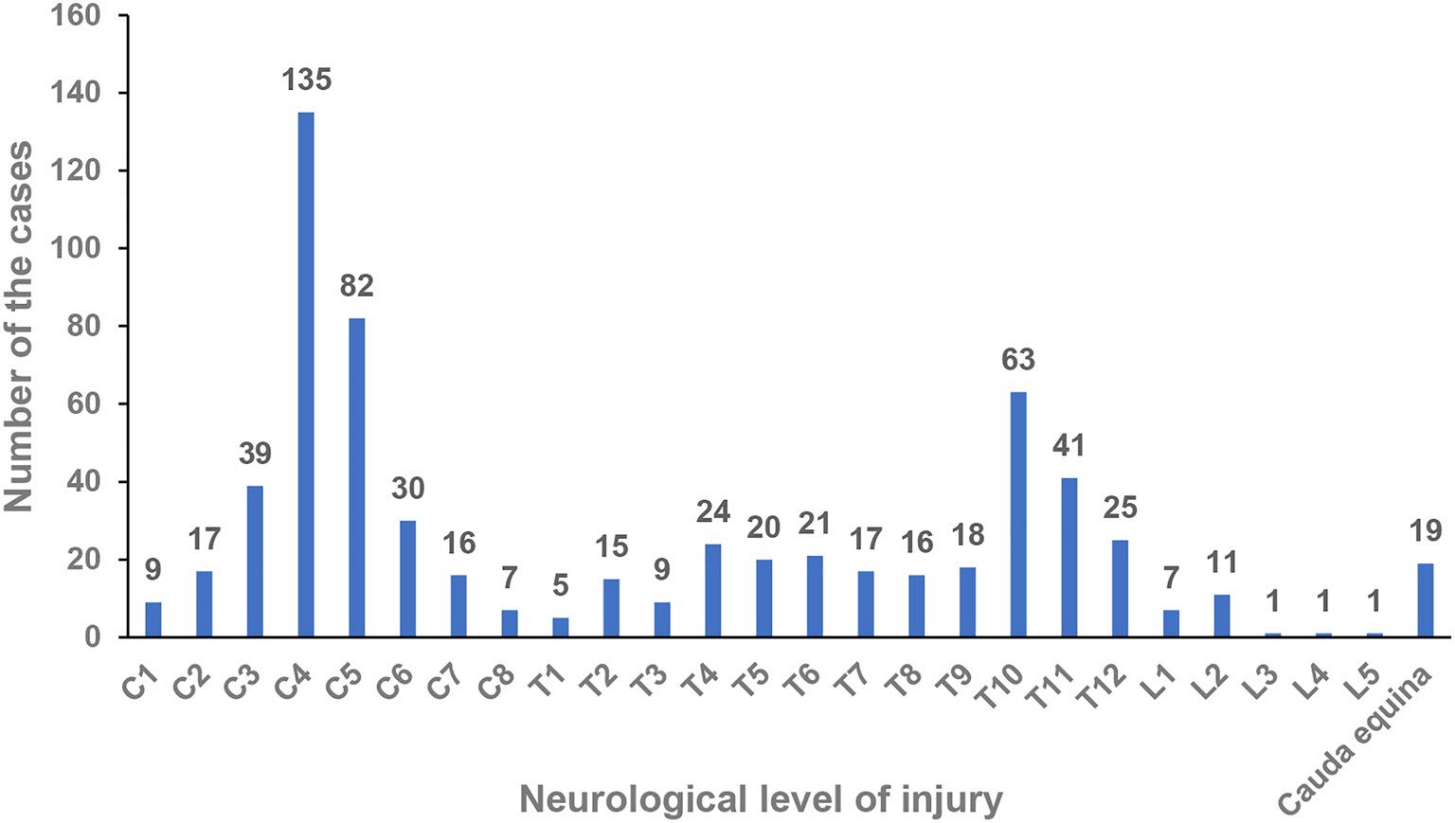
Distribution of TSCI level in all 649 cases.

The neurological level of MVC-induced SCIs in this study is shown in Table 2. Most cases had cervical SCI (*n* = 335, 51.6%) or thoracic SCI (*n* = 274, 42.2%). The most common cervical SCI involved cervical 4 and cervical 5, accounting for 64.8% of cervical SCI cases. Thoracic SCI (T10-T11) was the second most common neurological level of injury, which accounted for 16% of all cases (Fig. 2).

**Table 2:** Characteristics of 649 cases according to different level and the severity of the injury.

Variables	The level and the severity of the injury				Total	Statistics	*P*
	Complete tetraplegia	Incomplete tetraplegia	Complete paraplegia	Incomplete paraplegia			
Frequency (%)	130 (20%)	205 (31.6%)	226 (34.8%)	88 (13.6%)	649 (100%)	χ2 = 72.004	< 0.001
**Gender**						χ2 = 18.127	< 0.001
Male	108 (83.1%)	159 (77.6%)	145 (64.2%)	62 (70.5%)	474 (73%)		
Female	22 (16.9%)	46 (22.4%)	81 (35.8%)	26 (29.5%)	175 (27%)		
**Fracture level**							
C1-C2	2 (1.5%)	11 (5.4%)	3 (1.3%)	0 (0%)	16 (2.5%)		
C3-C7	109 (83.8%)	121 (59%)	5 (2.2%)	8 (9.1%)	243 (37.4%)		
T1-T10	19 (14.6%)	16 (7.8%)	88 (38.9%)	33 (37.5%)	156 (24%)		
T11-L2	6 (4.6%)	1 (0.5%)	133 (58.8%)	50 (56.8%)	190 (29.3%)		
L3-L5	0 (0%)	4 (2%)	18 (8%)	7 (8%)	29 (4.5%)		
S	0 (0%)	0 (0%)	4 (1.8%)	0 (0%)	4 (0.6%)		
No	18 (13.8%)	76 (37.1%)	25 (11.1%)	12 (13.6%)	131 (20.2%)		
**Surgery**						χ2 = 5.652	0.130
Yes	122 (93.8%)	188 (91.7%)	200 (88.5%)	75 (85.2%)	585 (90.1%)		
No	8 (6.2%)	17 (8.3%)	26 (11.5%)	13 (14.8%)	64 (9.9%)		
**Surgical approach**							
Anterior	82 (63.1%)	108 (52.7%)	72 (31.9%)	21 (23.9%)	283 (43.6%)	χ2 = 53.541	< 0.001
Posterior	17 (13.1%)	61 (29.8%)	128 (56.6%)	51 (58%)	257 (39.6%)	χ2 = 86.362	< 0.001
Anterior–posterior	23 (17.7%)	19 (9.3%)	0 (0%)	3 (3.4%)	45 (6.9%)	F = 48.873	< 0.001
**Degeneration of vertebrae**						F = 13.241	0.002
No	129 (99.2%)	193 (94.1%)	224 (99.1%)	88 (100%)	634 (97.7%)		
Yes	1 (0.8%)	12 (5.9%)	2 (0.9%)	0 (0%)	15 (2.3%)		
**Consequences**							
Spasticity	24 (18.5%)	65 (31.7%)	27 (11.9%)	17 (19.3%)	133 (20.5%)	χ2 = 26.357	< 0.001
Osteoporosis	22 (16.9%)	29 (14.1%)	42 (18.6%)	20 (22.7%)	113 (17.4%)	χ2 = 3.487	0.322
Neurogenic bladder	65 (50%)	93 (45.4%)	117 (51.8%)	44 (50%)	319 (49.2%)	χ2 = 1.858	0.602
**Complications**							
Intestinal dysfunction	48 (36.9%)	78 (38%)	88 (38.9%)	37 (42%)	251 (38.7%)		
Urinary infection	59 (45.4%)	65 (31.7%)	66 (29.2%)	19 (21.6%)	209 (32.2%)		
Neuralgia	33 (25.4%)	61 (29.8%)	66 (29.2%)	21 (23.9%)	181 (27.9%)		
Respiratory infection	23 (17.7%)	36 (17.6%)	16 (7.1%)	8 (9.1%)	83 (12.8%)		
Pressure ulcer	17 (13.1%)	14 (6.8%)	29 (12.8%)	7 (8%)	67 (10.3%)		
Deep vein thrombosis	9 (6.9%)	16 (7.8%)	30 (13.3%)	6 (6.8%)	61 (9.4%)		
Hypotension	22 (16.9%)	28 (13.7%)	6 (2.7%)	1 (1.1%)	57 (8.8%)		
Hyponatremia	9 (6.9%)	10 (4.9%)	1 (0.4%)	0 (0%)	20 (3.1%)		
**Combined injury**							
Limb fracture	23 (17.7%)	33 (16.1%)	56 (24.8%)	19 (21.6%)	131 (20.2%)	χ2 = 5.696	0.127
Pelvic fracture	2 (1.5%)	4 (2%)	11 (4.9%)	2 (2.3%)	19 (2.9%)	F = 3.920	0.260
Craniocerebral injury	18 (13.8%)	38 (18.5%)	32 (14.2%)	16 (18.2%)	104 (16%)	χ2 = 2.308	0.511
Rib fracture	12 (9.2%)	15 (7.3%)	59 (26.1%)	23 (26.1%)	109 (16.8%)	χ2 = 38.017	< 0.001
Visceral injury	32 (24.6%)	38 (18.5%)	84 (37.2%)	26 (29.5%)	180 (27.7%)	χ2 = 19.463	< 0.001

The frequencies of cervical SCI increased from 8.7 to 78.4% with increasing age. Interestingly, the frequencies of thoracic SCI decreased from 87 to 18.9% with increasing age. The distribution of cervical SCI and thoracic SCI in different age groups was significantly different (*P* < 0.001). Among those with a cervical SCI, the majority (34.6%) were in the 30–44 age group followed by the 45–59 age group (31%). Cases with thoracic SCI accounted for the majority (35.8%) in the 15–29 age group followed by the 30–44 age group (31%) (Table 1).

### Severity of injury

The percentage of cases who had complete injury (Grade A) with complete motor and sensory function damage below the injury site was 54.9%. Except for the ≥ 60 age group, the proportion of complete SCI was at a high level in all age groups (Table 1).

Table 2 shows the MVC-induced TSCIs at the neurological level and the extent of injury distribution. A total of 226 cases (34.8%) were complete paraplegia, followed by incomplete tetraplegia in 205 cases (31.6%), complete tetraplegia in 130 cases (20%) and incomplete paraplegia in 88 cases (13.6%), and significant differences in distribution were observed (*P* < 0.001, χ2 = 72.0). The frequency of tetraplegia (51.6%) was slightly higher than that of paraplegia (48.4%), and cases with incomplete tetraplegia accounted for 61.2%. In addition, cases with tetraplegia showed a larger sex ratio than those with paraplegia (3.9 vs 1.9, *P* < 0.001, χ2 = 649.0).

### Vertebral fractures and surgical treatment

Single vertebral fracture was found in 98.3% of the cases. The three most common fracture levels were C3–C7 (*n* = 121, 59%) in incomplete tetraplegia, and both T11–L2 (*n* = 133, 58.8%) and T1–T10 (*n* = 88, 38.9%) in complete paraplegia. In addition, SCI without fracture and dislocation accounted for 20.2%. Among those who experienced incomplete tetraplegia, 37.1% had injuries without fracture and dislocation (Table 2).

Table 3 shows that the most common fracture level in drivers and passengers was C3–C7, while that in pedestrians was T11–L2. Burst fractures were the most common type of fracture, accounting for 27% of those with vertebral fractures. The most common burst fractures were thoracic vertebrae burst fractures (37.9%), followed by cervical vertebrae burst fractures (34.2%), and lumbar vertebrae burst fractures (27.9%). In addition, 29 cases had compression fractures of the thoracic vertebrae.

**Table 3:** Characteristics of 533 cases caused by MVCs according to different subjects.

Variables	The different subjects			Total	Statistics	*P*
	Drivers	Passengers	Pedestrian			
Frequency (%)	214 (40.2%)	234 (43.9%)	85 (15.9%)	533 (100%)		
**Gender**					χ2 = 39.384	< 0.001
Male	186 (86.9%)	144 (61.5%)	54 (63.5%)	384 (72%)		
Female	28 (13.1%)	90 (38.5%)	31 (36.5%)	149 (28%)		
**Be left out of the car**					χ2 = 0.839*	0.360
Yes	24 (11.2%)	33 (14.1%)		57 (10.7%)		
No	190 (88.8%)	201 (85.9%)		391 (73.3%)		
**Vehicle type**						
Non-motor vehicle	76 (35.5%)	11 (4.7%)	4 (4.7%)	91 (17.1%)	χ2 = 85.882	< 0.001
Motorcycle	27 (12.6%)	10 (4.3%)	1 (1.2%)	38 (7.1%)	χ2 = 17.164	< 0.001
Passenger car	92 (43%)	193 (81.6%)	62 (72.9%)	347 (65.1%)	χ2 = 25.545	< 0.001
Commercial vehicle	9 (4.2%)	20 (7.7%)	10 (11.8%)	39 (7.3%)	χ2 = 6.056	0.048
Others	10 (4.7%)	0 (0%)	5 (5.9%)	15 (2.8%)	F = 15.401	< 0.001
**Fracture level**						
C1-C2	4 (1.9%)	6 (2.6%)	3 (3.5%)	13 (2.4%)		
C3-C7	78 (36.4%)	101 (43.2%)	16 (18.8%)	195 (36.6%)		
T1-T10	55 (25.7%)	61 (26.1%)	20 (23.5%)	136 (25.5%)		
T11-L2	51 (23.8%)	78 (33.3%)	36 (42.3%)	165 (31%)		
L3-L5	11 (5.1%)	13 (5.6%)	4 (4.7%)	28 (5.3%)		
S	2 (0.9%)	1 (0.4%)	1 (1.2%)	4 (0.8%)		
**Accident type**						
Rear-end	13 (6.1%)	25 (10.7%)		38 (7.1%)	χ2 = 3.059*	0.080
Vehicle collision	90 (42.1%)	101 (43.2%)		191 (35.8%)	χ2 = 0.056*	0.813
Rollover	30 (14%)	36 (15.4%)		66 (12.4%)	χ2 = 0.166*	0.684
High vehicle fall	30 (14%)	28 (12%)		58 (10.9%)	χ2 = 0.418*	0.518
Struck objects	25 (11.7%)	31 (13.2%)		56 (10.5%)	χ2 = 0.250*	0.617
Flat tire	3 (1.4%)	6 (2.6%)		9 (1.7%)	χ2 = 0.290*	0.590
Fall from the vehicle	23 (10.7%)	5 (2.1%)		28 (5.3%)	χ2 = 14.145*	< 0.001

*Except for the comparison of this group of data using Chi-square test of 4 lines data, the rest adopt Chi-square test of 2 lines X 3 lists data.

Most cases had received spinal surgery (*n* = 585, 90.1%), including laminoplasty, spinal decompression, fusion, and internal fixation, with only a small proportion (9.9%) with conservative treatment. Among those with cervical SCI, 92.5% received spinal surgery and the most common surgical approach was the anterior approach (*n* = 190, 61.3%), followed by the posterior approach (*n* = 78, 25.2%) and the anterior–posterior approach (*n* = 42, 13.5%). Forty-nine cases reported spinal stenosis (7.6%). Of the 75 cases with disc herniation, the percentage of cervical disc herniation was 92%. Fifteen cases had vertebral degeneration, with cervical degeneration accounting for 93.3%.

### Consequences and associated injuries

Spasticity, osteoporosis, and neurogenic bladder were long-term consequences of TSCI. Individuals with incomplete tetraplegia had the highest frequency of spasticity (*n* = 65, 31.7%). However, there was no difference in the distribution of the level and severity of injury in those with osteoporosis or neurogenic bladder (*P* = 0.322, χ2 = 3.487; *P* = 0.602, χ2 = 1.858). Of 649 individuals with TSCI, 310 (47.8%) sustained a total of 543 associated injuries. The most common associated injury was visceral injury (*n* = 180, 27.7%) (Table 2).

### Complications

Four hundred and thirty cases developed complications during hospitalization. The most common complication was intestinal dysfunction (*n* = 251, 38.7%), followed by urinary tract infection (*n* = 209, 32.2%) and neuralgia (*n* = 181, 27.9%) (Table 2). Those in the ≥ 60 age group had the highest frequency of complications (81.1%), followed by the 45–59 age group (71.7%). Those in the 1–14 age group had the lowest frequency of complications (56.5%). There was no significant difference in the distribution of cases with and without complications according to the different age groups (*P* = 0.065, χ2 = 8.8) (Table 1).

### Individuals role and etiology

The roles of individuals in MVCs, their frequencies, gender, vehicle type, fracture level, accident type, and other characteristics are summarized in Table 3. One hundred and sixteen records did not detail the role of the individual. Of the available patient roles in MVCs, the most common roles were passengers (234, 43.9%) and drivers (214, 40.2%). The percentage of pedestrians hit by vehicles was 15.9%. Passengers had the lowest male-to-female sex ratio (1.6), and the highest frequency of spine fractures (48%). Drivers had the highest male-to female sex ratio with 86.9% males and 13.1% females, and a lower frequency of being left out of the car compared with passengers, 11.2% and 14.1%, respectively. There was no significant difference in being left out of the car between the driver and passenger groups (*P* = 0.360, χ2 = 0.839).

Of all the traffic accidents included, vehicle collision was the main cause, accounting for 35.8%. Rollover was the second most common cause of MVCs, accounting for 12.4% on average. Other causes included high vehicle fall (10.9%), struck an object (10.5%), rear-end (7.1%), fall from vehicle (5.3%), and flat tire (1.7%). In addition, all pedestrians were hit by vehicles (Table 3).

### Vehicle type in accident

Table 4 shows the distribution of vehicle type involved in the traffic accidents. Collision accounted for 60% and falling down accounted for 21.1% of MVCs among non-motor vehicles. Collision accounted for 46% and falling down accounted for 24.3% of MVCs among motorcycles. Collision accounted for 50% and rollover accounted for 13.9% of MVCs among passenger cars, and collision accounted for 57.5% and rollover accounted for 15% of MVCs among commercial cars.

**Table 4:** Distribution of vehicle type in participants.

Accident type	Vehicle type			
	Non-motor vehicle	motorcycle	Passenger car	Commercial car
Rear-end	1 (1.1%)	2 (5.4%)	32 (9.2%)	3 (7.5%)
Collision	54 (60%)	17 (45.9%)	173 (50%)	23 (57.5%)
Rollover	3 (3.3%)	3 (8.1%)	48 (13.9%)	6 (15%)
High fall	9 (10%)	3 (8.1%)	39 (11.3%)	5 (12.5%)
Hit the object	4 (4.4%)	3 (8.1%)	46 (13.3%)	2 (5%)
Flat tire	0 (0%)	0 (0%)	8 (2.3%)	1 (2.5%)
Fall down	19 (21.1%)	9 (24.3%)	0 (0%)	0 (0%)
Total	90 (17.5%)	37 (7.2%)	346 (67.4%)	40 (7.8%)

## Discussion

A total of 649 cases with TSCI caused by MVCs were investigated over 10 years. Our findings showed that MVC-induced TSCI should not be neglected and preventive measures and intervention strategies should be improved.

In previous studies, MVCs were the leading cause of TSCI in developed countries^7,17,18^. In contrast, falls were the main cause of TSCI in developing countries^19,20^. A possible explanation for this may be the result of more cars per person in developed countries. With more and more drivers in China, the number of MVC-induced TSCIs has gradually increased. A considerably higher incidence of TSCIs of 23.7 per million was reported in Tianjin (2004–2008)^4^. In Heilongjiang province, the proportion of TSCIs caused by MVCs showed an increasing trend during 2009–2013^21^. In Beijing, MVCs had replaced falls as the leading cause of TSCI in a 2013 survey^22^. Of course, the car ownership is not the only reason. Safety training of driving, both for the drivers and the passengers are also very important.

Our data showed that the average age at the time of injury was 37.3 years old. Different regions of China and other countries have demonstrated different mean ages at TSCI, for example, Tianjin at 46.0 years old^4^, Turkey at 35.5 years old^23^, and Iran at 31.0 years old^24^. Despite the aging problem in China, the majority of drivers in China are young and middle aged. The frequency of MVC-induced TSCIs was highest in the 30–44 age group, followed by the 15–29 age group. These results are similar to those reported in Tianjin and Stockholm, Sweden^1,6^. This may be because most people obtain a driver's license as adults and own their first car after getting a job. Besides, our study showed that paraplegia account for 87% in the 1–14 age group, while quadriplegia (78.4%) was more likely to occur in elderly people aged 60 and above. People under 14 years of age were mostly motor complete injuries (Grade A or B, 78.3%), which may be because they were structed by motor vehicles as pedestrian while playing around without guardianship.

TSCI was more common in men than in women (ratio 2.7:1). The frequency of TSCI caused by MVCs increased with age in men, while women sustaining MVC-induced TSCI at a younger age. This may be related to different characteristics between men and women. Most male drivers tend to drive their vehicles at a high speed. Men tend to engage in high-risk lifestyles, such as alcohol drinking and driving while fatigued on highway^25^.

Our study indicated that about one quarter (26.3%) of participants were still unmarried when they were admitted to our hospital for rehabilitation after TSCI by MVCs. Most cases (62.9%) paid their hospitalization expenses at their own expense, which brings large economic burden to their family and society. More than four fifths of participants (81.7%) were discharged within one year. There was no significant difference in the distribution of rehabilitation length of stay and total cost among different age groups.

At a global level, MVCs involving motor vehicles, bicycles, and pedestrians account for the most part of SCIs^26^. In our study, passenger cars were the most common vehicles involved in accidents, including cars, off-road vehicles, and multi-purpose vehicles. Non-motor vehicles, such as bicycles, electric assist bicycles, and tricycles, were the second most common vehicles involved in MVCs. Rapid development of the automobile industry has led to a sustained increase in the number of motor vehicles over the past 5 years in China^21^. Collision and rollover occurred more frequently in passenger car accidents. Protecting vulnerable road users should also be further emphasized, particularly as substantial increases in the number of seriously injured pedal cyclists have been reported^27^.

Our study indicated that employed people were relatively more prone to TSCI than the unemployed. Up to 26.1% of cases were employed at the time of injury, followed by unemployed at 16%, which was different from previous reports^1,19^. Although the unemployed have a high rate of TSCI, the employed are more likely to be on the road due to their daily commutes and thus have a higher risk of traffic accidents^1^.

Speed limits are not strictly enforced, which can result in rear-end collisions or hitting pedestrians. Moreover, research shows that drivers are contributing factors to road traffic accidents (on average 99.3%)^28^. Drug use and alcohol intake by drivers were previously identified as risk factors for MVC-induced TSCI in several studies^29,30^. Unfortunately, we were unable to collect information on alcohol consumption or drug use prior to injury. As these factors are likely to contribute to a high proportion of MVC-induced TSCIs, we suggest improving traffic safety training and vehicle design, enforcing speed limits, and avoiding driving when fatigued or when alcohol has been consumed^11,21^.

The number of cases with tetraplegia was slightly higher than those with paraplegia, and was different from other developing countries^23,31^. Cases with tetraplegia require more care and have great difficulty in getting another job^32^. Three-fifths of cases had incomplete tetraplegia, due to degenerative changes in the vertebrae. The proportion of individuals who sustained a cervical injury due to MVCs was 51.6% in this study. This is in accordance with a previous study which reported that the cause of cervical injury was mainly due to road traffic accidents^5^. This can be attributed to a significant increase in C4-C5 lesions, which may lead to an increase in the frequency of respiratory infections. In 54.8% of cases, complete TSCI (Grade A) caused by MVCs was initially diagnosed, which was in accordance with the findings of Mrharaj (1996)^33^. Therefore, an investigation into the frequency, level and severity of MVC-induced TSCIs is important for appropriate medical care.

Of course, there are several limitations in this study. Firstly, variables including fracture level, surgical approach, degeneration of vertebrae, associated injuries, vehicle type, accident type, and role of all participants were manually recorded and extracted by researchers, which may be inaccurate or missing. One hundred and sixteen participants had no information on the type and role of traffic accidents, which accounted for 17.9% of the total number of cases. Secondly, the exact location of the injuries in all individuals is uncertain, and they may have obtained the injuries in different regions of China. Therefore, our study results might not be applicable to all persons with TSCI caused by MVCs. Thirdly, we did not collect information on pre-hospital deaths, which may have underestimated the severity of TSCIs caused by MVCs. Lastly, the method’s limitation was that the choice of methods itself means that confounders are likely to be part of our results.

## Conclusions

According to the characteristics of MVC-induced TSCIs, preventive strategies should be improved. The results of this study provide the epidemiological features of TSCI caused by MVCs based on the database of the CRRC from 2010 to 2019. Employed people were a high-risk population. Furthermore, collision was the leading cause of MVC-induced TSCI. The most common vehicles involved in MVCs were passenger cars and non-motor vehicles. The main causes of MVCs in passenger cars were vehicle collision and rollover, and the leading causes in non-motor vehicles were collision and fall from the vehicle. In addition to traffic accident management, the employed, passenger car drivers, cyclists, and males should be targeted in the prevention of MVCs. These findings can be used as a reference for policymakers in the prevention of MVC-induced TSCIs. Future investigations should also include the effects of environmental factors. In light of the increase in car owners, there is an urgent need for effective collision prevention programs to reduce MVC-induced TSCIs.

## Data Availability

The datasets used during the current study are available from the corresponding author on reasonable request.

## References

[1] Feng HY, Ning GZ, Feng SQ, Yu TQ, Zhou HX (2011). Epidemiological profile of 239 traumatic spinal cord injury cases over a period of 12 years in Tianjin China. J. Spinal Cord. Med..

[2] Kumar R (2018). Traumatic spinal injury: global epidemiology and worldwide volume. World Neurosurg..

[3] Yang NP (2008). The incidence and characterisation of hospitalised acute spinal trauma in Taiwan–a population-based study. Injury.

[4] Ning GZ (2011). Epidemiology of traumatic spinal cord injury in Tianjin China. Spinal Cord..

[5] Li J (2011). The epidemiological survey of acute traumatic spinal cord injury (ATSCI) of 2002 in Beijing municipality. Spinal Cord..

[6] Divanoglou A, Levi R (2009). Incidence of traumatic spinal cord injury in Thessaloniki, Greece and Stockholm, Sweden: a prospective population-based study. Spinal Cord..

[7] Pickett GE, Campos-Benitez M, Keller JL, Duggal N (2006). Epidemiology of traumatic spinal cord injury in Canada. Spine (Phila Pa 1976).

[8] O'Connor PJ, Brown D (2006). Relative risk of spinal cord injury in road crashes involving seriously injured occupants of light passenger vehicles. Accid Anal Prev.

[9] Wang H (2016). Incidence and pattern of traumatic spinal fractures and associated spinal cord injury resulting from motor vehicle collisions in China over 11 years: an observational study. Med. (Baltim.).

[10] Wang MC, Pintar F, Yoganandan N, Maiman DJ (2009). The continued burden of spine fractures after motor vehicle crashes. J. Neurosurg. Spine.

[11] Liu, J., Liu, H. W., Gao, F., Li, J. & Li, J. J. Epidemiological features of traumatic spinal cord injury in Beijing, China. *J. Spinal Cord. Med.* pp. 1–7 (2020). 10.1080/10790268.2020.1793505 PMC8986294 32703104

[12] Biering-Sørensen F (2017). International spinal cord injury core data set (version 2.0)-including standardization of reporting. Spinal Cord.

[13] Kirshblum SC (2011). Reference for the 2011 revision of the international standards for neurological classification of spinal cord injury. J. Spinal Cord Med..

[14] Manworren RC, Stinson J (2016). Pediatric pain measurement, assessment, and evaluation. Semin. Pediatr. Neurol..

[15] Hunt A (2004). Clinical validation of the paediatric pain profile. Dev. Med. Child Neurol..

[16] Suraseranivongse S (2001). Cross-validation of a composite pain scale for preschool children within 24 hours of surgery. Br. J. Anaesth..

[17] van den Berg ME, Castellote JM, Mahillo-Fernandez I, de Pedro-Cuesta J (2010). Incidence of spinal cord injury worldwide: a systematic review. Neuroepidemiology.

[18] O'Connor RJ, Murray PC (2006). Review of spinal cord injuries in Ireland. Spinal Cord.

[19] Gur A (2005). Characteristics of traumatic spinal cord injuries in south-eastern Anatolia, Turkey: a comparative approach to 10 years' experience. Int. J. Rehabil. Res..

[20] Agarwal P, Upadhyay P, Raja K (2007). A demographic profile of traumatic and non-traumatic spinal injury cases: a hospital-based study from India. Spinal Cord.

[21] Chen R (2017). Current epidemiological profile and features of traumatic spinal cord injury in Heilongjiang province, Northeast China: implications for monitoring and control. Spinal Cord.

[22] Hua R (2013). Analysis of the causes and types of traumatic spinal cord injury based on 561 cases in China from 2001 to 2010. Spinal Cord.

[23] Karacan I (2000). Traumatic spinal cord injuries in Turkey: a nation-wide epidemiological study. Spinal Cord.

[24] Rahimi-Movaghar V (2009). Prevalence of spinal cord injury in Tehran Iran. J Spinal Cord Med.

[25] Mirzaeva L, Gilhus NE, Lobzin S, Rekand T (2019). Incidence of adult traumatic spinal cord injury in Saint Petersburg Russia. Spinal Cord.

[26] Sekhon LH, Fehlings MG (2001). Epidemiology, demographics, and pathophysiology of acute spinal cord injury. Spine (Phila Pa 1976).

[27] Beck B (2017). Road safety: serious injuries remain a major unsolved problem. Med. J. Aust..

[28] Ramadani N (2017). Public health profile of road traffic accidents in Kosovo 2010–2015. Open Access Maced J Med Sci.

[29] Lenehan B (2012). The epidemiology of traumatic spinal cord injury in British Columbia Canada. Spine (Phila Pa 1976).

[30] Sabre L (2012). High incidence of traumatic spinal cord injury in Estonia. Spinal Cord.

[31] Hoque MF, Grangeon C, Reed K (1999). Spinal cord lesions in Bangladesh: an epidemiological study 1994–1995. Spinal Cord.

[32] Wyndaele M, Wyndaele JJ (2006). Incidence, prevalence and epidemiology of spinal cord injury: what learns a worldwide literature survey?. Spinal Cord.

[33] Maharaj JC (1996). Epidemiology of spinal cord paralysis in Fiji: 1985–1994. Spinal Cord.

